# Inactivation of the Progesterone Receptor in Mx1+ Cells Potentiates Osteogenesis in Calvaria but Not in Long Bone

**DOI:** 10.1371/journal.pone.0139490

**Published:** 2015-10-02

**Authors:** Zhendong A. Zhong, Weihua Sun, Haiyan Chen, Hongliang Zhang, Nancy E. Lane, Wei Yao

**Affiliations:** 1 Center for Musculoskeletal Health, Department of Internal Medicine, University of California Davis Medical Center, Sacramento, CA, 95817, United States of America; 2 Department of Emergency Medicine, The Second Xiangya Hospital of Central-South University, Hunan, Changsha, China; Kyungpook National University School of Medicine, REPUBLIC OF KOREA

## Abstract

The effect of progesterone on bone remains elusive. We previously reported that global progesterone receptor (PR) knockout mice displayed high bone mass phenotype, suggesting that PR influences bone growth and modeling. Recently, Mx1+ cells were characterized to be mesenchymal stem cell-like pluripotent Cells. The aim of this study was to evaluate whether the PR in Mx1+ cells regulates osteogenesis. Using the Mx1-Cre;mT/mG reporter mouse model, we found that the calvarial cells exhibited minimal background Mx1-Cre activity prior to Cre activation by IFNα treatment as compared to the bone marrow stromal cells. IFNα treatment significantly activated Mx1-Cre in the calvarial cells. When the PR gene was deleted in the Mx1-Cre;PR-flox calvarial cells *in vitro*, significantly higher levels of expression of osteoblast maturation marker genes (RUNX2, Osteocalcin, and Dmp1) and osteogenic potential were detected. The PR-deficient calvariae exhibited greater bone volume, especially in the males. Although Mx1-Cre activity could be induced on the bone surface *in viv*o, the Mx1+ cells did not differentiate into osteocytes in long bones. Bone volumes at the distal femurs and the bone turnover marker serum Osteocalcin were similar between the Mx1-Cre;PR-flox mutant mice and the corresponding wild types in both sexes. In conclusion, our data demonstrates that blocking progesterone signaling via PRs in calvarial Mx1+ cells promoted osteoblast differentiation in the calvaria. Mx1+ was expressed by heterogeneous cells in bone marrow and did not differentiate into osteocyte during long bone development *in vivo*. Selectively inactivating the PR gene in Mx1+ cells affected the membrane bone formation but did not affect peripheral skeletal homeostasis.

## Introduction

There is a sexual dimorphism in bone mass acquisition; males typically achieve greater peak bone mass (PBM) and greater bone size than females. Females experience an acceleration of bone loss due to declining estrogen levels with menopause and aging [1]. Thus, females are at greater risks of developing osteopenia and osteoporosis. Sex hormones (sex steroids/gonadal steroids) synthesized from cholesterol in the gonads and adrenal glands have been considered to be the primary mediators of sexual dimorphism in the bone. The biosyntheses of these hormones are closely related, which makes it difficult to separately study individual hormones [2]. Taking advantage of the fact that sex hormones act on target cells by binding to members of the nuclear hormone receptor superfamily [3], scientists have studied how estrogen and androgens regulate bone homeostasis by manipulating their corresponding receptors [4, 5]. Although sex hormone receptor knockout models do not necessarily recapitulate the phenotypes of sex hormone deficiency, the functions of estrogen receptors (ERs) and androgen receptors (ARs) on bone homeostasis have been extensively studied using animal models with germ-line or tissue-specific knockouts. These genetically modified mouse models have provided crucial information about the complex functions of sex hormone receptors in relation to bone homeostasis. For example, estrogen receptors display either stimulatory or inhibitory functions on bone formation depending on ligand availability and target skeletal location [5].

The effect of progesterone, another important sex hormone, on bone homeostasis remains unclear. Most of the studies that have evaluated the effect of progesterone on bone have utilized either agonists or antagonists in preclinical or clinical studies, and the results are conflicting. However progesterone agonist and antagonist treatments may alter the levels of other hormones, such as estrogen, androgens, and follicle-stimulating hormone (FSH), which makes it difficult to dissect the true progesterone responses [6, 7]. There are two major PR isoforms—PR-B and PR-A—in mammals. PR-A and PR-B exhibit different biological functions that are specific to particular cell types and promoter contexts [3]. Although majority of research indicates that PR expression alteration or gene mutation is closely related to tumorigenesis and cancer progression, PR dysfunction is also associated with cardiovascular defects (aortic aneurysm), neurological defects (migraine, vertigo) and reproductive conditions (endometriosis, infertility) [8–12]. Our research group and others have reported a high-bone-mass phenotype in global progesterone receptor knockout (PRKO) mice age two to 12 months of age, and this phenotype appears to result from a greater bone formation rate in females and a reduced bone resorption rate in males measured at three months of age [13, 14]. These data suggest that progesterone signaling through PR may suppress bone mass acquisition in mice. However, it is unclear how PRs affect bone homeostasis; PRs are differentially expressed in a wide range of tissues, including those of the reproductive system and central nervous system. Because PRs are expressed in the osteoblasts, and PRKO bone marrow stromal cells exhibit increased osteogenic potential *in vitro* [15–17], we hypothesized that PRs may directly regulate osteoblastic cells. In this study we utilized the Cre-Lox system to selectively delete both PR isoforms from osteoprogenitor cells to investigate its function in skeletal system.

Mx proteins are the main effectors of the antiviral innate immune response mediated by type I interferon (IFN I) [18]. The Mx1 promoter is active in bone marrow stromal cells and has been used to drive Cre recombinase expression to study gene function in bone and other tissues [19, 20]. Recently, Mx1+ cells were characterized to be mesenchymal stem cell-like pluripotent Cells, which can differentiate into an osteoblastic lineage *in vivo*, but cannot differentiate into chondrocytes and adipocytes [21, 22]. The purpose of this study was to use Mx1-Cre to drive PR gene knockout in osteoblastic lineage *in vivo* and *in vitro* to study the effects of PRs on osteogenesis.

## Results

### 1. Specificity and induction efficacy of Mx1-Cre *in vivo* and *in vitro*

**Fig 1:**
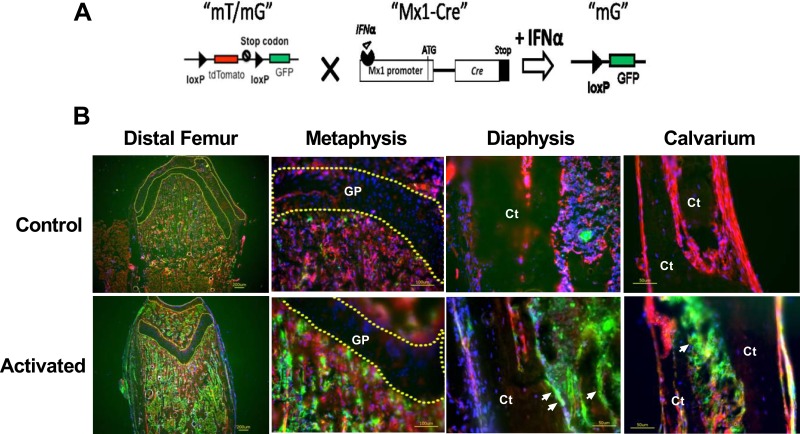
Characterization of Mx1-Cre in the long bones and calvariae *in vivo*. **(A)** A schematic diagram showing that mT/mG is crossed to Mx1-Cre and that IFNα can activate Cre expression to delete the tdTomato (red fluorescent) cassette and initiate GFP (green fluorescent, “mG”) expression. **(B)** Mx1-Cre;mT/mG mice at the age of 5 weeks were injected intraperitoneally with PBS (control) or pI-pC (activated). The distal femurs and calvariae were collected for cryosection after three days. The nuclei were stained with DAPI (blue). The arrows indicate bone surface GFP expression in the pI-pC-treated bones. The dotted lines circle the cartilage regions in the distal femur. GP, growth plate cartilage; Ct, cortical bone.

The Mx1 promoter is silent in healthy mice but can be induced in multiple cell lineages by the administration of interferon α, interferon β, or synthetic double-stranded RNA (e.g., poly I:C, “pI-pC”) [23]. There is ~1% background recombination in mice that are not treated with interferon, and this level varies with tissue type presumably due to the numbers of interferon-responsive cells that are present or the availability of interferon in each organ [http://jaxmice.jax.org/strain/005673.html]. However, the Mx1-cre promoter’s specificity in bone tissue has not been elucidated in detail. We generated a Mx1-Cre;mT/mG reporter model to study the activity of Mx1-Cre in bone tissues and primary cultured osteoblasts. The mT/mG reporter mice constitutively express the tomato protein (red) unless they have been exposed to Cre recombinase. Exposure to Cre recombinase results in the deletion of the tomato cassette and the induction of GFP (green) expression [24] (Fig 1A).

We treated 1-month-old Mx1-Cre;mT/mG double transgenic mice with pI-pC, and collected the calvariae and distal femurs for cryosection (Fig 1B). We observed small numbers of cells within the bone marrow that were GFP-positive, which corresponded to Cre activity. In contrast, the calvariae exhibited relatively fewer GFP-positive cells prior to pI-pC treatment. One week after pI-pC treatment, we observed a robust induction of GFP expression in the bone marrow and bone surfaces of the distal femurs and calvariae. The growth plate region remained GFP-negative prior to and after the pI-pC treatment, indicating Mx1-Cre did not affect the chondrocytes *in vivo* (Fig 1B).

**Fig 2:**
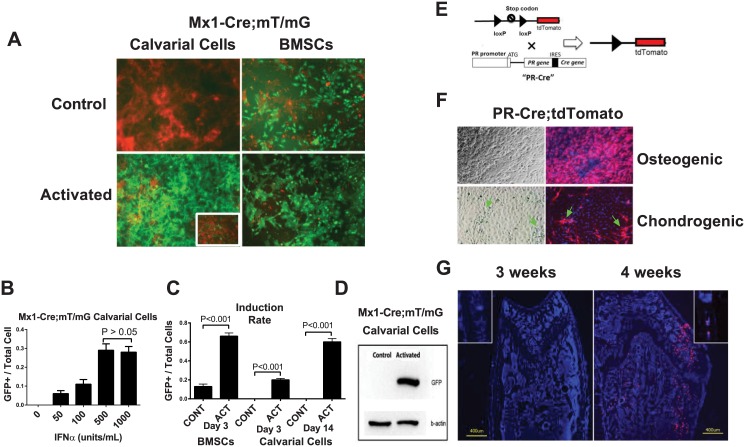
Comparison of BMSCs and calvarial cells in terms of Mx1-Cre activation and PR expression *in vitro*. (**A**) BMSCs or calvarial cells were obtained from Mx1-Cre;mT/mG double transgenic mice and treated with IFNα (500 units/mL) for three days. Fluorescent images were taken three days (BMSCs) or 14 days (Calvarial cells) after the IFNα treatment. The image of calvarial cells three days post IFNα treatment is shown in the insert. The levels of Cre activation were quantitated by GFP (green) expression. (**B**) A quantitative histogram showing the induction rates (the GFP+ cells versus the total cells) of the BMSCs and calvarial cells shown in Figure A. CONT, control; ACT, activated. (**C**) Western blotting was performed to detect GFP protein in the total cell lysates from the calvarial cells with (activated) or without (control) IFNα treatment on day 3. β-actin was used as an internal control. (**D**) Mx1-Cre;mT/mG calvarial cells were treated with different concentrations of IFNα for three days. The ratios of GFP+ cells to total cells under each IFNα concentration were quantified (A). A PR-Cre;tdTomato schematic diagram showing that the Cre gene is inserted downstream of the endogenous PR gene after an internal ribosome entry site (IRES) sequence such that Cre is expressed simultaneously with PR. When Cre is expressed (together with PR), it recombines loxp sites to remove the stop codon before the tdTomato cassette and activates tdTomato (red fluorescence) expression. (E) A diagram of PR-Cre; tdTomato. The tdTomato expression can be activated following the upstream stop codon removal by Cre, which is expressed simultaneously with endogenous PR gene. So that PR+ cells will express tdTomato (red fluorescent). (**F**) PR-Cre;tdTomato calvarial cells were differentiated into osteoblasts with osteogenic media or chondrocytes with chondrogenic media for 14 days. The majority of the cells cultured in the osteogenic medium were red. However, when the cells were cultured in the chondrogenic medium, only a small percentage of the fibroblast-like cells (arrows) turned red. The nuclei were stained with DAPI (blue). (G) Distal femurs or calvariae (inserts) from 3-week-old or 4-week-old PR-Cre;tdTomato mice were sectioned for fluorescent microscopy. The nuclei were stained with DAPI (blue). The red fluorescence indicated PR promoter activity.

Calvarial cells and bone marrow stromal cells (BMSCs) are two commonly used sources of osteoprogenitor or pre-osteoblast cultures. *In vitro* experiments were mostly performed in cells derived from male mice to exclude the potential estrogen and progesterone effects during menstrual cycles in females. We collected BMSCs from 1-month-old male Mx1-Cre:mT/mG mice and calvarial cells from 3-day-old Mx1-Cre:mT/mG pups, and treated the cells with IFNα (500 units/mL) for 72 hours to activate the Mx1-Cre promoter. Similar to its expression *in vivo*, we observed substantial basal Cre activity in the BMSCs (13% were GFP-positive prior to activation), and 66% of the BMSCs became GFP-positive after IFNα treatment (Fig 2A and 2B). In contrast, the calvarial cells exhibited extremely low background Cre activity prior to IFNα treatment. Following IFNα treatment, approximately 30% of the calvarial cells became GFP-positive three days after IFNα treatment, and ~60% became GFP-positive two weeks after IFNα treatment (Fig 2B). We also determined that the minimal IFNα dose required to induce robust Mx1-Cre activity in the calvarial cells was 500 units/mL (Fig 2B). These findings were confirmed by western blot; i.e., no GFP band was detected prior to activation, and a GFP band was present three days after induction in the Mx1-Cre;mT/mG calvarial cells (Fig 2C). Thus, we concluded that the calvarial cells were superior to the BMSCs for *in vitro* studies due to the low basal Mx1-Cre activity prior to activation.

### 2. PR knockout in Mx1+ calvarial cells and calvariae increased osteoblast differentiation *in vitro*

To confirm the PR expression in the calvarial cells, we first generated a PR-Cre; tdTomato model (Fig 2E) in which Cre is driven by the endogenous promoter of the PR gene to activate tdTomato (red fluorescence) expression in the cells that express or have expressed PRs. We isolated calvarial cells from the PR-Cre; tdTomato double transgenic pups and differentiated the cells into either osteoblasts or chondrocytes. Fourteen days after differentiation, the majority of the osteogenic cells were tdTomato-positive, while only a small percentage of fibroblast-like cells that were typically and morphologically not chondrocyte-like cells were tdTomato-positive in the chondrogenic culture (Fig 2F). These results suggest that in ex vivo culture systems, the majority of osteoblastic cells express the PR during osteogenesis but not during chondrogenesis. Additionally, PR expression was not observed *in vivo* until the mice were 4 weeks of age (Fig 2G).

**Fig 3:**
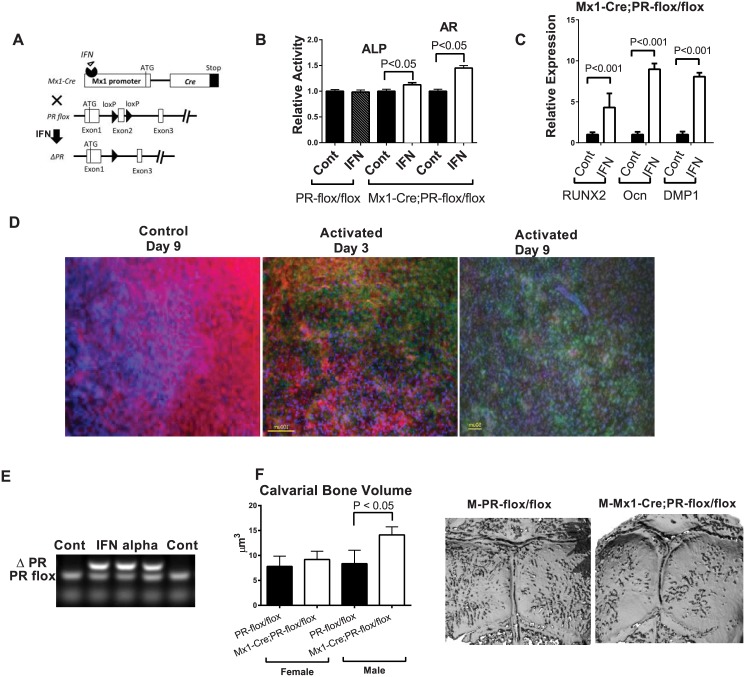
PR inactivation in the Mx1+ calvarial cells and calvariae *in vitro*. (**A**) A schematic diagram showing that PR-flox is crossed to Mx1-Cre. Mx1-Cre can be activated by IFNα to delete exon 2 of the PR gene to generate PR mutants (ΔPR). (**B**) Mx1-Cre;PR-flox/flox calvarial cells were treated with IFNα (500 units/mL) or without IFNα (control) for three days. The cells were then differentiated into osteoblasts in osteogenic medium without IFNα for 14 days. The relative expressions of RUNX2, Osteocalcin (Ocn) and DMP1 were evaluated by real-time PCR at day 14 and normalized to endogenous β-actin. (**C**) The cells were collected for alkaline phosphatase (ALP) activity assays on day 10 and alizarin red staining (AR) on day 21. The optical density (OD) values were normalized to the corresponding total protein concentrations. Calvarial cells from the PR-flox/flox (without Cre) mice were used as a negative control to exclude the effect of INFα itself. (**D**) The calvariae obtained from Mx1-Cre;mT/mG double transgenic pups exhibited significant numbers (~40%) of cells that became GFP-positive after three days of IFNα (500 units/mL) treatment, and significantly more cells (~80%) became GFP-positive after an additional six days of culture without IFNα. (**E**) Genomic DNA was isolated from PR-flox/flox calvarial cells three days after IFNα treatment, and subjected to PR allele-specific PCR. The deleted PR band (ΔPR) indicated Cre-mediated DNA recombination. (**F**) Five-weeks-old PR-flox/flox or Mx1-Cre;PR-flox/flox mice were injected with pI-pC. Calvariae were collected five months later for microCT analysis for bone volume and (G) representative calvarial images from microCT scans.

We next generated an inducible PR conditional knockout mouse model by crossing Mx1-Cre and PR-flox mice. Following IFNα treatment, the Mx1 promoter drives Cre recombinase expression, which recombines the loxP sites and deletes exon 2 of the PR gene resulting in PR inactivation in the Mx1+ cells (Fig 3A). We then collected calvarial cells from the Mx1-Cre;PR-flox double transgenic pups and treated the cells with or without IFNα (500 units/mL) for three days. The calvarial cells were then differentiated into osteoblasts in osteogenic media without IFNα treatment. RNA was collected at 0, 7 and 14 days post differentiation. Real-time PCR revealed significantly greater osteogenic marker gene expression in the IFNα-treated cells on day 14. Specifically, the RUNX2, osteocalcin (Ocn) and DMP1 gene expressions were increased by 4-fold, 9-fold, and 9-fold, respectively, compared with the control groups (Fig 3B). Additionally, the Mx1-Cre-mediated PR knockout significantly increased osteoblast activity as measured by the alkaline phosphatase activity (ALP) at 10 days post-differentiation and mineralized nodule formation (alizarin red, AR) at 21 days post-differentiation (Fig 3C). IFNα treatment by itself had no effect on osteoblast differentiation in the PR-flox/flox (no-Cre control) calvarial cells (Fig 3C). These data suggest that inactivation of the PR gene in the Mx1+ calvarial cells accelerated osteoblasts maturation.

To study the functions of PRs in the calvarial bone tissue, we developed an *ex vivo* calvarium organ culture system. First, we wanted to confirm the activation of Mx1-Cre in the *ex vivo* cultured calvarium. The Mx1-Cre;mT/mG double transgenic calvariae were induced with IFNα-containing BGJb medium (500 units/mL) for three days and were then cultured in BGJb medium without IFNα thereafter. A significant proportion (~40%) of calvarial cells appeared GFP-positive after three days of IFNα treatment, and significantly more cells (~80%) became GFP-positive by nine days (Fig 3D), indicating that the Mx1+/GFP+ calvarial cells were capable of proliferating. In separate experiment, we obtained calvariae from the Mx1-Cre;PR-flox/flox double transgenic mice and treated the calvarial tissue following a similar protocol. Using PR allele-specific PCR, we were able to detect the deleted PR band after PR-flox calvarial cells were treated with IFNα (Fig 3E). In line with these data from the *in vitro* experiments, we found that bone volume of the calvariae in male Mx1-Cre;PR-flox knockout mice was 67% higher than their WT littermates (P < 0.05) at five months after PR was selectively knocked out in the Mx1+ cells from five weeks of age (Fig 3F and 3G). Calvarial bone thickness was similar between the WT and Mx1-Cre;PR-flox/flox mice in both sexes (data on file).

### 3. Mx1+ cell fate mapping in long bones

**Fig 4:**
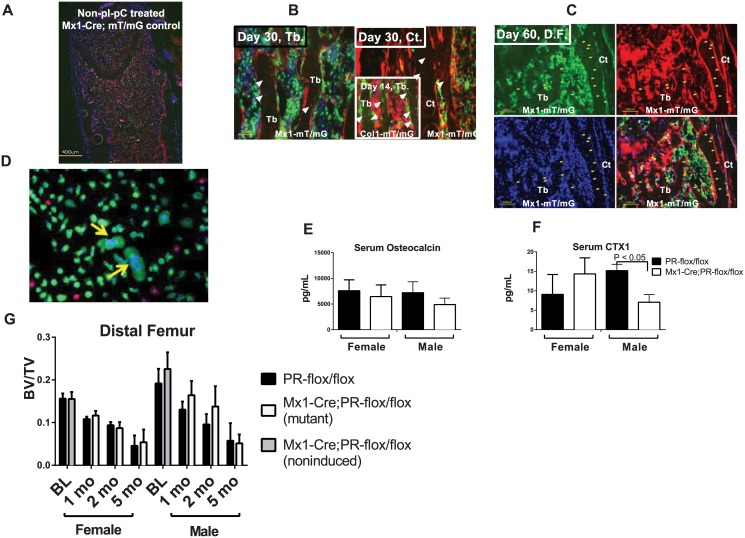
Skeletal phenotypes of Mx1-Cre-driven PR inactivation *in vivo*. (A) A distal femur from non-pI-PC treated Mx1-Cre;mT/mG mouse. (B—C) Mx1-Cre;mT/mG mice were injected with pI-pC intraperitoneally to induce Cre at one month of age and then sacrificed one (B) or two (C) months later. The distal femurs (D.F.) were collected and sectioned to observe the GFP (green) and tdTomato (red) fluorescence. Green indicates the Mx1+ cells that expressed Cre, and red indicates the Cre-negative cells. The nuclei were stained with DAPI (blue). The femoral trabecular bones from the Col1a1-CreERT2;mT/mG mice that received 4 days of tamoxifen injections were used as a positive control and are shown in the insert in (B). The white arrows indicate the green osteocytes that were observed in the trabecular bone in the Col1a1-mT/mG mice but were absent in the trabecular or cortical bone of the Mx1-mT/mG mice (the white arrowheads indicate the GFP-/tdTomato+ osteocytes). There were no green osteocytes observed in the Mx1-mT/mG bones on either day 30 or 60. (D) Bone marrow cells were collected from 1-month-old Mx1-Cre;mT/mG mice. Some multinuclear cells turned green indicating Mx1-Cre activation in these cells (yellow arrows). (E) Serum osteocalcin and (F) serum CTX1 levels were measured by ELISA two months post pI-pC injection (n = 8/group). (G) Five-week-old male and female Mx1-Cre;PR-flox/flox or PR-flox/flox (control) mice were injected with pI-pC intraperitoneally to induce Cre activity. Baseline microCT scans (BL) of the distal femurs were performed on five-week-old mice without receiving the pI-pC injection. The animals were sacrificed at one, two or five months post-pI-pC injection, and the hind limbs were collected for microCT analysis (n = 8/group).

We next asked whether Mx1+ cells represent “osteoprogenitors” that can terminally differentiate into osteocytes in long bones *in vivo*. We aged the pI-pC-treated Mx1-Cre;mT/mG mice to evaluate whether their Mx1+/GFP+ cells could eventually differentiate into osteocytes. We found some green positive cells were detected in mice that were not treated with pI-pC, suggesting a baseline leaky nature of Mx1-Cre in bone marrow (Fig 4A). After 30 or 60 days post pI-pC treatment (Mx1 activation), Mx1+ cells were found very heterogeneous expressed by both the hematopoietic and mesenchymal lineages and while most of Mx–1+ cells were observed within bone marrow, some were observed at the trabecular bone surface. We failed to detect any GFP+ osteocytes that were embedded in the trabecular or cortical bone (Fig 4B and 4C). We used Col1a1-CreERT2;mT/[mG as a positive control and we identified some GFP+ osteocytes two weeks after tamoxifen treatment in these tamoxifen-inducible Col1a1-CreERT2;mT/mG mice (Fig 4B, insert). Bone marrow cells culture suggested Mx1 was expressed by multinuclear cells (Fig 4E). These data indicate that the Mx1+ cells were expressed by mononuclear and multinuclear cells within bone marrow, osteoblasts at bone surface but were not able to differentiate into osteocytes in the long bones *in vivo*.

### 4. Mx1-Cre-driven PR knockout did not affect long bone phenotype

The Mx1-Cre;PR-flox/flox mice were viable, exhibited body weights and sizes that were similar to those of their wild-type littermates and exhibited no obviously abnormal behavior (data not shown). To characterize the *in vivo* skeletal phenotypes of the Mx1-Cre;PR-flox mice, we treated the Mx1-Cre;PR-flox/flox mice with pI-pC at five weeks of age to delete the PR gene. PR-flox/flox (without Cre) mice were used as controls. At one, two and five months post-treatment, the distal femurs were collected and subjected to microCT analyses. A baseline scan was performed at five weeks of age before the pI-pC treatment. Serum osteocalcin levels between wild types and mutants were similar (Fig 4D) and serum CXT1 level (Fig 4E) was lower in the Mx1-Cre;PR-flox/flox mice as compared to their WT littermates at two months post pI-pC treatment. Additionally, we did not observe significant BV/TV changes at one, two and five-months post-PR inactivation in Mx1+ cells in both sexes (Fig 4F).

## Discussion

In this study, we found that the Mx1 promoter activity could be robustly induced on the femoral bone surface. However, we did not observe Mx1+ expression in the chondrocytes or differentiation into the osteocytes that were embedded within the bone matrix in long bones. In contrast to the *in vivo* Mx1 activity, using an *in vitro* culture system, we did observe Mx1+ calvarial cells proliferating and differentiating into osteoblasts. Our findings regarding Mx1-Cre are in line with those of a previous publication in which the authors used a single pulse of pI-pC and *in vivo* imaging to show that Mx1 labeled a majority of osteogenic progenitors and a fraction the mature osteoblasts but not the osteocytes. Moreover, Mx1+ cells have been shown to be capable of differentiating into osteocytes during fracture healing [21]. Taken together, these findings suggest that Mx1+ cells derived from calvarium contribute to osteoblastogenesis in intramembranous bone formation but may not participate in endochondral bone formation. Mx1-Cre specificity may vary depending on the ossification processes and the microenvironment. Mx1 seems to primary participate in the intramembranous ossification in the calvarium and plays minimum role in the endochodral ossification in long bones. However, additional studies are required to support our observations.

In the present study, calvarial cells were characterized as a good model for Mx1-Cre *in vitro* because they exhibited minimal basal Mx1-Cre activation prior to IFNα treatment, and the Cre activity could be robustly activated by IFNα. However, the Mx1-Cre in BMSCs exhibited significant basal activation prior to IFNα treatment. As IFN secretion increases with age and microbe exposure [25], the basal Mx1 activity in the BMSCs from 1-month-old mice might have been induced by endogenous IFN.

We found that the calvarial cells exhibited a significantly greater potential to differentiate into osteoblasts after the PR expression was ablated from the Mx1+ cells. The observation of significantly greater levels of osteogenic markers 14 days after differentiation indicates that PRs might play a role in the later stages of osteoblast differentiation. These observations are consistent with our previous finding that BMSCs from PRKO mice exhibit increased osteoblastic differentiation *in vitro* [13]. In contrast, a number of *ex vivo* studies have reported that supplementation of the culture medium with progesterone can stimulate cell proliferation and osteoblastic differentiation possibly through the action of insulin-like growth factors (IGFs). This anabolic effect of progesterone on osteoblast differentiation is more robust in female-derived osteoblasts than those from males [26–29]. PR-mediated progesterone signaling acts through specific progesterone response elements (PRE) within the promoter regions of target genes to regulate transcription, and this process is referred to as the “classical” mechanism. Moreover, some of progesterone’s effects may also result from the “non-genomic” effects of PRs that are mediated by PR [30]. One example of progesterone’s non-genomic effects is the activation of the extracellular signal–regulated kinase (ERK)–mitogen-activated protein kinase (MAPK) pathway; this pathway is critical for osteoblast differentiation and skeletal development [31]. Based on our results and those of others [30], we speculate that PR inactivation in Mx1+ cells might not block progesterone’s non-genomic effects or recapitulate progesterone antagonism. In our study, PR inactivation did not exhibit an effect on osteoblast differentiation that was exactly opposite to the reported effect of progesterone supplementation *in vitro*. Additionally, the timing and levels of PR expression in osteoprogenitor cells vary depending upon the source of the derived osteoprogenitor cells. PR expression was detected in *in-vitro*-cultured calvarial cells from 3-day-old pups. However, PR expression in mouse bones (including the calvarium) *in vivo* was not detectable until one month of age (data on-file). This lack of PR expression may have contributed to the discrepancy that we observed between the *in vitro* and *in vivo* data. Moreover, Mx1+ cells are found to be expressed by both the hematopoietic as well as the mesenchymal lineage cells in bone marrow. Mx1-Cre is active in multinuclear cells and PR deletion in Mx1+ cells might affect the function of osteoclast as suggested by lower bone resorption in the male Mx1-Cre; PR-flox mice. Most importantly, Mx1+ cells do not differentiate into osteocytes in long bones, which may explain the lack of a peripheral skeletal phenotype in the Mx1-Cre;PR-flox mutant mice *in vivo*. Additional studies using different Cre drivers are underway to pinpoint the effects of PRs on the different stages of osteoblastogenesis, bone development, and the PR-mediated downstream effectors.

In summary, PR inactivation in the Mx1+ cells derived from calvarium enhanced osteoblast differentiation *in vitro and in vivo*. In contrast, Mx1-Cre is leaky and expressed by heterogeneous cell populations within bone marrow. The Mx1+ cells did not differentiate into osteocytes in the long bones *in vivo*, which perplexed the interpretation of the peripheral bone phenotype in our Mx1-Cre-driven PR knockout model. Mx1-Cre may not be a best cre to be used to study the contribution of “mesenchymal” lineage to osteogenesis.

## Experimental Procedures

### Mouse Lines

The PR-flox mice were obtained from Dr. John Lydon at Baylor College of Medicine. Briefly, a targeting vector designed to replace part of exon 2 of the PR gene with a selectable marker was used to create a strain of mice carrying a conditional null PR allele [32]. Mx1-Cre, Col1a1(2.3 kb)-CreERT2, and mT/mG transgenic mice were from the Jackson Laboratory. The mT/mG reporter mouse possesses loxp sites at both sides of a tdTomato (mT) cassette and expresses red fluorescence in all tissues. When bred with Cre-expressing mice, the resulting offspring can express the downstream-enhanced green fluorescent protein (mG) due to the deletion of the mT cassette in the Cre-expressing tissues. PCR-based strategies were then used to genotype the mouse genomic DNA (details available upon request). After intraperitoneal injections of 250 μg of pI-pC per mouse per day for three consecutive days, all the animals were healthy (8 per group) showing no adverse events to injections, and sacrificed 1, 2 and 5 months post-injection, and the hind limbs were collected. All animal work was done in compliance with the guiding principles of the “Care and Use of Animals”. Mice were housed in the animal facility under closely controlled environmental conditions (12-hour light/dark cycle, room temperature 22°C), and fed ad libidum (food and water). The animal protocol was approved by the Institutional Animal Care and Use Committee of the University of California Davis (animal protocol #18322). All surgeries were performed under anesthesia, and all efforts were made to minimize animal suffering.

### MicroCT measurements

The right distal femur from each animal was scanned and analyzed using VivaCT 40 (Scanco Medical, Bassersdorf, Switzerland) with a voxel resolution of 10 μm in all three spatial dimensions and a mono-energetic (70 KeV) X-ray source. For the distal femurs, we evaluated 200 slices that were situated approximately 0.2 mm from the distal end of the growth plate. The slides covered a total metaphyses tissue volume of 2–3.5 mm^3^ for each scan and were used to obtain the cancellous bone volume/total volume (BV/TV) ratio [13]. The calvariae was collected from PR-flox/flox and Mx1-Cre; PR-flox/flox mice at 4 months post pI-pC injection. Scans and evaluations were imitated from the anterior suture and included 30 slices (0.3 mm) at the frontal region and 200 slices (2mm) at the parietal region but excluded the squamous, sphenoid and occipital bones (Fig 3G).

### Calvarium and Calvarial Cell Culture

Calvariae (5 per group) were collected from 3-day old pups. A cut was made through the sagittal suture to separate the calvarium into two halves. One half was used as a control, and the other half was used for the IFNα treatment. BGJb medium (Life Technologies, Carlsbad, CA) supplemented with 0.1% bovine serum albumin and 100 U/mL each of penicillin, streptomycin and amphotericin was used for the calvarium culture. The calvarial tissues were cultured in 24-well plates with 8-μm hanging PET inserts (Millipore, Billerica, Massachusetts) as described previously [33]. The calvarial cells were isolated from the calvariae by serial digestion at 37°C with constant agitation in 0.2 mg/ml collagenase type I (Worthington, Lakewood, NJ). The digestion solution was changed and collected an additional five times. Digestions 2–6 (containing the osteoprogenitor and osteoblasts cells) were centrifuged and washed with α-MEM containing 10% FBS and 1% penicillin/streptomycin and cultured for 48 hours at 37°C before use in the further experiments.

IFNα was used to activate Mx1-Cre *in vitro*. In brief, the calvariae or calvarial cells were incubated in medium containing IFNα (R&D System, Minneapolis, MN) for three days, after which the IFNα medium was replaced with fresh IFNα-free medium. A dose-response study was first performed using the Mx1-Cre;mT/mG calvarial cells to optimize the IFNα dose. The induction rate was determined by the ratio of the GFP-positive cell number to the total cell number based on 250 cells from five different areas in the wells. The calvarial cells were differentiated into osteoblasts in osteogenic media (changed every two days) containing 50 μg/ml ascorbic acid and 10 mM β-glycerol phosphate (βGP).

### Quantitative Real-time RT-PCR Analysis

Total RNA was extracted from the cells using RNeasy (Qiagen, Venlo, Netherlands). The extracted RNA was used for cDNA synthesis via reverse transcriptase using a OneStep RT-PCR Kit and random primers (QIAGEN, Venlo, Netherlands). The cDNA samples were subjected to PCR analysis using Fast SYBR PCR Master Mix and an ABI 7500 Real-time PCR system (Applied Biosystems, NY). The primers used were as follows: RUNX2 (TGGCTTGGGTTTCAGGTTAG, CCTCCCTTCTCAACCTCTAATG); Osteocalcin (TGTGACGAGCTATCAAACCAG, GAGGATCAAGTTCTGGAGAGC); and the DMP1 primers (HP226170) were ordered from the ORIGENE company. The mRNA level of each gene was normalized to the endogenous β-actin level.

### Staining and Immunohistochemistry

The bones from the Mx1-Cre;mT/mG mice were collected, fixed in 4% formaldehyde with 10% sucrose (W/V) at 4°C overnight, and embedded in Optimal Cutting Temperature (O.C.T.) medium. Cryosections were cut using a CryoJane tape transfer system for a cryostat (Leica Biosystem, IL) and were and observed using a fluorescent microscope. For the quantification of the alkaline phosphatase (ALP) activity, the cell lysate was incubated with One-Step NBT-BCIP solution (Thermo Scientific, Waltham, MA) at 37°C and read at 570 nm. The optical density (OD) values were normalized to the corresponding protein concentrations. For the Alizarin Red staining, the cells were rinsed with PBS and fixed for one hour with 100% ethanol and stained for 30 minutes with Alizarin Red Solution (40 mM solution prepared in dH2O, pH 4.1). The Alizarin Red was resolved in a solution of 20% methanol and 10% acetic acid in water and read on a spectrophotometer at 450 nm. The quantity was normalized to the protein content. A BCA assay (Thermo Scientific, Waltham, MA) was performed to quantify the protein concentration. The calvarium tissues were fixed in 4% paraformaldehyde for 24 hours and then decalcified in 10% EDTA for two days. The decalcified tissues were paraffin-embedded, and 4-μm sections along the sagittal suture were adhered to glass slides as previously described [33]. Immunohistochemistry was performed with anti-RUNX2 antibody (Cell Signaling, Danvers, MA.), and an ImmPACT DAB kit (Vector Labs, Burlingame, CA) was used to develop the signal.

### Statistical Analysis

Results are expressed as mean ± SEM. One-way ANOVA followed by the Bonferroni test was used to measure statistically significant differences between groups. A value of P < 0.05 was considered statistically significant. Data were analyzed using the Gaphpad Prism software package (La Jolla, CA).

## Abbreviations

ALP: alkaline phosphatase
BMSC: bone marrow stromal cell
Cre: Cre recombinase
ER: estrogen receptor
FBS: fetal bovine serum
GFP: green fluorescent protein
IFNα: interferon alpha
IHC: immunohistochemistry
microCT: micro-computed tomography
Ocn: osteocalcin; pI-pC, poly I:C
PR: progesterone receptor
